# Clinical validation of the gastrointestinal NET grading system: Ki67 index criteria of the WHO 2010 classification is appropriate to predict metastasis or recurrence

**DOI:** 10.1186/1746-1596-8-65

**Published:** 2013-04-22

**Authors:** Takeshi Yamaguchi, Takahiro Fujimori, Shigeki Tomita, Kazuhito Ichikawa, Hiroyuki Mitomi, Kazuya Ohno, Yosuke Shida, Hiroyuki Kato

**Affiliations:** 1 Department of Surgical and Molecular Pathology, Dokkyo Medical University, 880 Kitakobayashi, Mibu, Shimotsuga, Tochigi, Japan; 2 First Department of Surgery, Dokkyo Medical University, 880 Kitakobayashi, Mibu, Shimotsuga, Tochigi, Japan; 3 Department of Gastroenterology, Shizuoka City Shizuoka Hospital, 10-93, Otemachi, Aoi-ku, Shizuoka, Japan

## Abstract

**Background:**

In the WHO 2010 classification, the neuroendocrine tumors (NETs) are subdivided by their mitotic index or Ki67 index into either G1 or G2 NETs. Tumors with a Ki67 index of <2% are classified as G1 and those with 3—20% are classified as G2. However, the assessment of tumors with Ki67 index of greater than 2% and less than or equal to 3% is still unclear. To resolve the problem, we validated the Ki67 index criteria of gastrointestinal NETs of the WHO 2010 classification.

**Methods:**

The medical records of 45 patients who were pathologically diagnosed as having NET G1/G2 of the gastrointestinal tract were analyzed retrospectively. According to the WHO 2010 classification, Ki67 index were calculated. Computer-assisted cytometrical analysis of Ki67 immunoreactivity was performed using the WinRooF image processing software. Receiver operating characteristic (ROC) curves were generated to determine the best discriminating Ki67 index. To clarify the assessment of tumors with Ki67 index between 2—3%, the calculated cutoff of Ki67 index was evaluated using Fisher’s exact test.

**Results:**

ROC curve analysis confirmed that 2.8% was the best Ki67 index cutoff value for predicting metastasis or recurrence. The sensitivity of the new Ki67 index cutoff was 42.9%, and the specificity was 86.8%.

**Conclusions:**

Division of NETs into G1/G2 based on Ki67 index of 3% was appropriate to predict metastases or recurrences. The WHO grading system may be the most useful classification to predict metastases or recurrences.

**Virtual Slides:**

The virtual slide(s) for this article can be found here: http://www.diagnosticpathology.diagnomx.eu/vs/1553036118943799

## Background

Since S. Oberndorfer proposed the term “carcinoid” in 1907 [1], the origins of neuroendocrine tumors (NETs) of the gastrointestinal tract as well as the malignancy of these tumors have been attracting the attention of clinicians [2-6]. After investigation by prognostic or diagnostic procedures, based on a wealth of evidence, the 2000 edition of the World Health Organization (WHO) classification provided a rational approach to the nomenclature and classification of NETs of the digestive system [7]. This system identified NETs as well differentiated endocrine tumors (WDET), well differentiated endocrine carcinomas (WDEC), and poorly differentiated endocrine carcinomas (PDEC) [8]. In 2010, a revised version of the WHO classification appeared. The new classification defines the entire group of tumors as neuroendocrine neoplasms (NENs), which have been confirmed to arise from the neuroendocrine cell system. NENs are shared with marker proteins of neuroendocrine cell system [7,9,10], and they are further categorized into neuroendocrine carcinomas (NECs) and NETs. NECs are morphologically similar to small cell carcinoma and large cell carcinoma of the lung, while NETs encompass neoplasms that were previously termed “carcinoid” or “atypical carcinoid” [7,9,10]. NETs are subdivided by their mitotic index or Ki67 index into either G1 or G2 NETs. This revised classification is a simple and useful grading system based on the proliferative activity. However, the assessment of tumors with Ki67 index of greater than 2% and less than or equal to 3% is still unclear. Despite this, due to large inter-observer differences in mitotic counts, the validity and reproducibility of Ki67 index are clearly superior to those of the mitotic index [11]. Tumors with a Ki67 index of <2% are classified as G1 and those with 3—20% are classified as G2. The aim of this study was to evaluate whether this grading system can predict metastasis or recurrence, to validate the Ki67 index criteria of gastrointestinal NETs of the WHO 2010 classification, and to especially clarify the uncertainty in assessment of tumors with Ki67 index between 2—3%. We performed computer-assisted cytometrical analysis of Ki67 immunohistochemistry (IHC), which was established in several of our past studies [12,13], using the WinRooF image processing software (Mitani Corp., Tokyo, Japan).

## Methods

### Study cases and tissue samples

The medical records of 45 patients who were pathologically diagnosed as having NET G1/G2 of the gastrointestinal tract were analyzed retrospectively. They were diagnosed at Dokkyo Medical University and its associated institutions between January 2003 and June 2012. Five cases were obtained by biopsy, 21 cases by endoscopic resection, and 19 cases by surgical resection. All cases were re-diagnosed and classified according to the criteria of the WHO 2010 classification. No case contained adenomatous component or any other lesion with NETs [14]. Cases with multiple tumors and tumors arising from the appendix were excluded. Histological diagnoses of all cases were confirmed by the pathological report, and neuroendocrine differentiation was confirmed immunohistochemically using antibodies directed against chromogranin A and synaptophysin. This study was performed with the approval of the ethics committee of each institution, and informed consent was obtained from all patients.

### Immunohistochemical staining for Ki67

Immunohistochemical staining for Ki67 was performed with a LSAB-2 kit (LSAB2 System-HRP; DAKO, Carpinteria, CA, USA) as described previously [15,16]. The 4-μm thick sections were placed on slides, deparaffinized, and dehydrated. They were then placed in 0.01 M citrate buffer (pH 6.0) and treated by microwave heating (400 W, 95°C; MI-77; Azumaya, Tokyo, Japan) for 40 minutes to facilitate antigen retrieval. Then, the sections were pretreated with 0.3% H2O2 in methanol at room temperature to quench endogenous peroxidase activity. This was followed by blocking with Protein Block Serum-Free (Dako, USA) for 30 minutes, and incubation with anti-Ki67 antibody (1:50 clone MIB-1; Dako, Japan) for 1 hour. Thereafter, the sections were incubated with biotinylated secondary antibody for 15 minutes, washed with PBS, and treated with peroxidase-conjugated streptavidin for 20 min. Finally, the sections were visualized by incubating in 3, 3′-diaminobenzidine tetrahydrochloride with 0.05% H2O2 (Liquid DAB + Substrate Chromogen System; Dako, USA) for 3 min and then counterstained with Carazzi’s hematoxylin.

### Evaluation of immunohistochemical Ki67 expression

**Figure 1:**
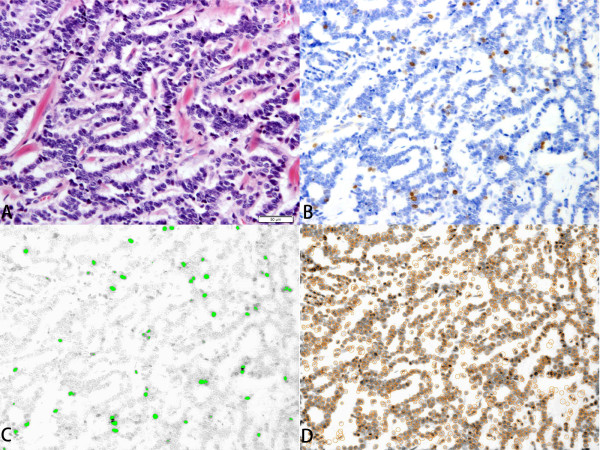
**Images of a case of NET.** This case is rectal NET with multiple liver and lung metastasis, and its Ki67 index is 2.8%. **A:** Hematoxylin and eosin staining. **B:** Immunohistochemical findings for Ki-67. **C:** Image of WinROOF. Count the Ki67 positive cells by B (blue) image of enhanced contrast for RGB color separation. **D:** Image of WinROOF. Count all tumor cells by R (red) image of enhanced contrast for RGB color separation.

Stratified sampling procedure was performed according to the WHO 2010 classification [17,18]. Ki67 indices were calculated as a percentage of Ki67 positive cells in 500—2000 cells that were counted in areas of strongest nuclear labeling (“hot spots”). Interactive virtual microscopy was performed to get standardized digital images [19]. Images were captured at ×15—×30 magnification, and computer-assisted cytometrical analysis of Ki67 immunoreactivity was performed using the WinRooF image processing software (Mitani Corp., Tokyo, Japan) [12,13,20-22]. In order to maximally differentiate the target cells from the adjacent ones, the margins of the nuclei were identified by enhanced contrast for RGB separation (Figure 1). B (blue) image with the “Separate Cells” function was the easiest to count Ki67 positive cells, and R (red) image with the “Separate Circular Figure” function was the easiest to count all tumor cells. Size and shape of tumor cells was manually calibrated, and the non-tumor cells were eliminated with a touch pen by introducing a liquid crystal touch panel.

### Statistical analysis

Statistical analysis was performed using R (version 2.15.0). For the analysis of risk factors for metastasis or recurrence, sex, age, location of tumor, resection methods, size, Ki67 index, and grade of WHO classification were evaluated using Fisher’s exact test or Welch two sample t-test. Receiver operating characteristic (ROC) curves were generated to determine the best discriminating tumor size and Ki67 index. The cutoffs for predicting metastasis or recurrence by each component were defined by the values with the highest accuracy that maximized the Youden index (sensitivity + specificity-1) [23,24]. To clarify the assessment of tumors with Ki67 index between 2—3%, the calculated cutoff of the Ki67 index was evaluated using Fisher’s exact test. Differences were considered to be statistically significant when P <0.05.

## Results

### Clinical characteristics

Among the 45 NET patients, seven cases (15.6%) showed evidence of metastasis or recurrence. Three patients had lymph node metastases, one had liver metastases, one had liver and lymph node metastases, one had liver and lung metastases, and another one had local recurrence. All 45 patients were divided into two groups: group A included those who had metastases or recurrences and group B were those who did not. There was no difference between the two groups with respect to the ratio of men to women, mean age, resection method, invasion depth, and location of the primary tumor. Metastases or recurrences occurred only from primary duodenal (2 cases of 10, 20.0%) and rectal (5 cases of 29, 17.2%) tumors. The patient characteristics are outlined in Table 1. ROC curve analysis confirmed that 20.5 mm was the best tumor size cutoff value for predicting metastasis or recurrence (area under the curve = 0.623). The sensitivity of the analysis was 33.3%, and the specificity was 100.0%. Although there was no difference between two groups with regard to mean tumor size, when using this cutoff value, tumors larger than 20.5 mm were predictive of metastasis or recurrence (p = 0.019).

**Table 1:** Clinical characteristics of patients with neuroendocrine tumors

	**Metastases or recurrences**		
	**+ (Group A, n = 7)**	**- (Group B, n = 38)**	**p value**
Gender			0.39
Male	4	28	
Female	3	10	
Mean age	58.4 (41—75)	61.9 (38—86)	0.50
Location			1
Stomach	0	4	
Duodenum	2 (20.0%)	8	
Small intestine	0	1	
Colon	0	1	
Rectum	5 (17.2%)	24	
Tumor invasion			0.16
M	0	1	
SM	4	28	
MP	2	3	
SS	1	1	
Unknown	0	5	
Resection methods			0.34
Biopsy	0	5	
Endoscopic resection	2	19	
Surgical resection	5	14	
Mean tumor size (mm)	12.9 (3.5—27)	7.4 (1.5—15)	0.26

Group A: the cases with metastases or recurrences.

Group B: the cases without metastases or recurrences.

Statistically significant difference (<0.05) by Fisher’s exact test or Welch two sample t-test.

### Ki67 IHC in NET

**Figure 2:**
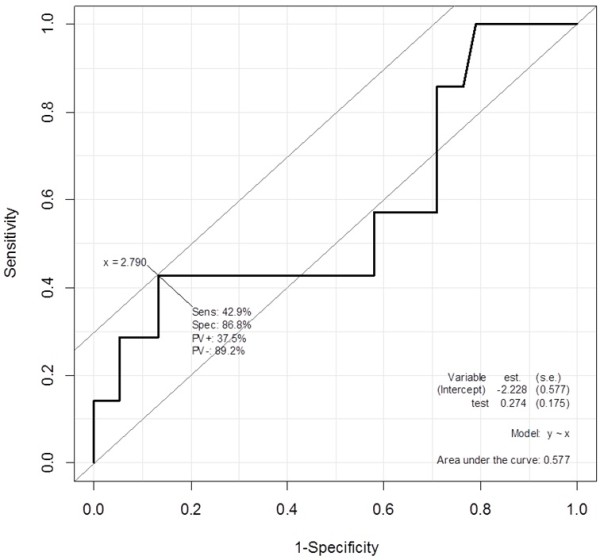
**The ROC curve analysis to determine the best discriminating Ki67 index.** Sens: sensitivity, Spec: specificity, PV+: positive predictive value,PV-: negative predictive value.

No statistical difference was found between the two groups with regard to mean Ki67 index (Group A: mean 2.83%, range 0.38—10.43%; Group B: mean 1.39%, range 0—6.44%). According to the WHO 2010 classification, all cases were classified into G1 and G2 based on the Ki67 index of 2% or 3% (Table 2), but there was no difference. Therefore, to determine the best discriminating Ki67 index, ROC curve analysis was performed (Figure 2), which determined that 2.8% was the best Ki67 index cutoff value for predicting metastasis or recurrence (area under the curve = 0.577). The sensitivity of the new Ki67 index cutoff was 42.9%, and the specificity was 86.8% (Table 3). Although there was no statistical difference in the new cutoff value of Ki67 index, compared with 2% (positive predictive value = 25.0%, negative predictive value = 87.9%), the new cutoff (positive predictive value = 37.5%, negative predictive value = 89.2%) was more appropriate for prediction of metastasis or recurrence. In clinical settings, a cutoff value of 3% may be better for dividing the tumors into G1/G2. If the cases were classified based on 3%, the sensitivity was 28.6% and the specificity was 89.5%.

**Table 2:** Analysis of Ki67 index, dividing into G1/G2 according to the WHO 2010 classification

	**Metastases or recurrences**		
	**+ (Group A, n = 7)**	**- (Group B, n = 38)**	**p value**
Mean Ki67 index (%)	2.83 (0.38—10.43)	1.39 (0—6.44)	0.35
Grade			
G1 (Ki67 index ≦2%)	4	29	
G2 (Ki67 index >2%)	3	9	0.36
Grade			
G1 (Ki67 index ≦3%)	5	34	
G2 (Ki67 index >3%)	2	4	0.23

Group A: the cases with metastases or recurrences.

Group B: the cases without metastases or recurrences.

Statistically significant difference (<0.05) by Fisher’s exact test or Welch two sample t-test.

**Table 3:** Dividing by new cutoff value of Ki67 index

	**Metastases or recurrences**		
	**+ (Group A, n = 7)**	**- (Group B, n = 38)**	**p value**
Ki67 index ≦2.8%	4	33	
Ki67 index >2.8%	3	5	0.09

Group A: the cases with metastases or recurrences.

Group B: the cases without metastases or recurrences.

Statistically significant difference (<0.05) by Fisher’s exact test.

### Separating analysis by location of the primary tumor

Metastases or recurrences only occurred from primary duodenal and rectal tumors, so the separating analysis was performed according to each location. Among the patients of NETs of the duodenum, 2 of 10 cases had metastases or recurrences. Grade, tumor size, and invasion depth were not predictive of metastasis or recurrence in this group (Table 4). In invasion deeper than submucosa, duodenal NET G1 showed an equivalent incidence of metastases as NET G2. On the other hand, among the patients of rectal NET, 5 of 29 cases had metastases or recurrences, and there were statistical differences in invasion depth and Ki67 index (Table 5). Division of rectal NETs into G1/G2 was predictive of metastasis or recurrence, which was different from that of NETs in the duodenum.

**Table 4:** Separating analysis in the primary of duodenum

	**Metastases or recurrences**		
	**+ (Group A, n = 2)**	**- (Group B, n = 8)**	**p value**
Mean tumor size (mm)	15.5 (5—26)	7.63 (2—15)	0.59
Tumor invasion			1
M	0	1	
SM	2	5	
MP	0	2	
SS	0	0	
Mean Ki67 index (%)	0.56 (0.40—0.73)	1.55 (0.34—4.29)	1

Group A: the cases with metastases or recurrences.

Group B: the cases without metastases or recurrences.

Statistically significant difference (<0.05) by Fisher’s exact test or Welch two sample t-test.

**Table 5:** Separating analysis in the primary of rectum

	**Metastases or recurrences**		
	**+ (Group A, n = 5)**	**- (Group B, n = 24)**	**p value**
Mean tumor size (mm)	11.5 (3.5—27)	7.11 (1.5—15)	0.13
Tumor invasion			<0.01
M	0	0	
SM	2	22	
MP	2	0	
SS	1	0	
Un known	0	2	
Mean Ki67 index (%)	3.73 (0.38—10.44)	1.24 (0.22—6.44)	0.022

Group A: the cases with metastases or recurrences.

Group B: the cases without metastases or recurrences.

Statistically significant difference (<0.05) by Fisher’s exact test or Welch two sample t-test.

## Discussion

S. Oberndorfer noted in 1907 that the multiple unusual tumors in the small bowel were distinct clinical entities and proposed the term “carcinoid” (“carcinoma-like”), emphasizing in particular their benign features [1,2,25]. However, malignancy of this tumor group has been confirmed based on metastatic or survival rates in long term studies [26-28]. Some studies have discussed the tumor origins and suggested that these neoplasms may arise from a type of endocrine cells, known as amine precursor uptake and decarboxylation (APUD) cells [2-4]. Although the APUD cells were initially suggested to be derived from neural crest cells, it is now generally recognized that gastroenteropancreatic APUD cells probably arise from the endoderm [2,29]. Tumor definition was initially based on typical morphological characteristics, and subsequent studies demonstrated argyrophilia-staining properties of the tumors using silver impregnation techniques, such as the Grimelius method [2,30-33]. Moreover, the neuroendocrine origin of the tumors was definitively identified and assessed in detail using stains for neuroendocrine differentiation markers, chromogranin A, and/or synaptophysin [10,34]. After these investigations by prognostic or diagnostic procedures, the term "carcinoid" has been regarded as a misnomer [28]. Since these tumors are considered as cancers of the neuroendocrine system, a more adequate term “neuroendocrine tumors” is now widely used. Based on these lines of evidence, the WHO 2000 classification provided a rational approach to the nomenclature and classification of NETs of the digestive system [7], which subdivided them into WDET, WDEC, and PDEC [8]. NETs have historically been classified according to the foregut, midgut, or hindgut derivation, and they have been indicated the prognostic differences. Some studies have also demonstrated the prognostic potential of IHC and TNM classification [5,6]. Since long-term follow up studies have indicated the malignancy of these tumors, in 2010, a revised version of the WHO classification appeared [7]. The new classification defines the entire group of tumors as NENs, which have been confirmed to arise from the neuroendocrine cell system since they are shared with marker proteins of this system [9,10]. NENs are further categorized into NECs and NETs. NECs are morphologically similar to small cell carcinoma and large cell carcinoma of the lung, while NETs encompass neoplasms that were previously termed “carcinoid” or “atypical carcinoid” [7,9,10]. The NETs are subdivided by their mitotic index or Ki67 index into either G1 or G2 NETs. This revised classification is a simple and useful grading system based on the proliferative activity. However, the assessment of tumors with Ki67 index that is greater than 2% and less than or equal to 3% is still unclear. Despite this, the inter-observer differences in mitotic counts are larger than that of Ki67 index, and moreover, it is difficult to scan routinely at least 50 high power fields (HPFs) (1 HPF = 2 mm2), that is required in the WHO 2010 classification for evaluation of the mitotic index. Thus, the validity and reproducibility of Ki67 index are superior to those of the mitotic index [11]. Tumors with a Ki67 index of <2% are classified as G1 while those of 3—20% are classified as G2. Because one cutoff value is used to divide continuous values into two groups, we validated the Ki67 index criteria of gastrointestinal NETs of the WHO 2010 classification with the aim of clarifying the assessment of tumors with Ki67 index between 2—3%. We performed computer-assisted cytometrical analysis of Ki67 IHC, which was established in several of our past studies [12,13], using the WinRooF image processing software. Although it was not considered generally, a concordance study using other softwares indicated that digital image analysis and manual count were highly concordant and were acceptable standards for Ki67 assessment [21]. Furthermore, in our experience, the number of cells could be more accurately and objectively counted with some devices. In this study, several problems occurred which were similar to those in our previous studies. One of them was that the non-tumor cells, such as lymphocytes or histocytes, occasionally showed positivity for Ki67. To resolve this, the non-tumor cells were eliminated with a touch pen by introducing a liquid crystal touch panel as our previous reports. After that, we evaluated whether the grading system could accurately predict metastasis or recurrence. Our results confirmed that 2.8% (or approximately 3%) is the best Ki67 index cutoff value for predicting metastasis or recurrence. If the cases were classified based on 3%, the sensitivity was 28.6% and the specificity was 89.5%, which is meaningful for predicting metastasis or recurrence because of the high specificity. On the other hand, due to the low sensitivity, metastases may be observed not only in G2 but also in G1 regardless of biological malignancy. In recent studies, the location of the primary tumor, size, invasion depth, and multiplicity of tumors were associated with metastasis or recurrence [26,35], and in our study, it was confirmed that tumor size was correlative. It was also reported that the presence of Circulating Tumor Cells (CTCs) with cellular expression of epithelial cell adhesion molecule (EpCAM) was more predictive of clinical outcomes than the WHO grading system [36]. However, the methods to isolate CTCs for molecular characterization are still being developed, and there are few studies regarding CTCs in patients with NETs [36,37]. Thus, at present, the WHO grading system may be the most useful classification to predict metastasis or recurrence. In summary, division of NETs into G1/G2 based on Ki67 index of 3% was appropriate to predict metastasis or recurrence (positive predictive value = 33.3%, negative predictive value = 87.2%). To the best of our knowledge, this is the first study to validate the numerical criteria of the WHO 2010 classification of NETs and to indicate the appropriateness of the Ki67 index. Further investigation is required in order to support this finding.

## Conclusions

ROC curve analysis confirmed that 2.8% is the best Ki67 index cutoff value for predicting metastases or recurrences. Division of NETs into G1/G2 based on Ki67 index of 3% was appropriate to predict metastases or recurrences. The WHO grading system may be the most useful classification to predict metastases or recurrences thus far.

## Abbreviations

NET: Neuroendocrine tumor; NEN: Neuroendocrine neoplasm; NEC: Neuroendocrine carcinoma; WHO: The World Health Organization; WDET: Well differentiated endocrine tumor; WDEC: Well differentiated endocrine carcinoma; PDEC: Poorly differentiated endocrine carcinoma; IHC: Immunohistochemistry; ROC: Receiver operating characteristic; APUD: Amine precursor uptake and decarboxylation; HPF: High power field; EpCAM: Epithelial cell adhesion molecule; CTC: Circulating Tumor Cell.

## Competing interests

The authors declare that they have no competing interests.

## Authors’ contributions

TY collected clinical data, evaluated the immunohistochemical stainings, performed the statistical analyses and drafted the manuscript. KO assisted with the design of the study and the collection of clinical data. TF conceived the study, was involved in the design and immunohistochemical analysis, and edited the manuscript for intellectual content. ST, KI, HM, YS and HK were involved in the design of the study and pathological diagnosis. All authors read and approved the final manuscript.
